# Candida antifungal resistance: It's not just about hospital acquisition and antifungal consumption

**DOI:** 10.1016/j.onehlt.2026.101641

**Published:** 2026-10-31

**Authors:** Ronan Adler Tavella, Daniela Debone, Simone Georges El Khouri Miraglia, Arnaldo Lopes Colombo

**Affiliations:** a ARIES, Antimicrobial Resistance Institute of São Paulo, São Paulo, Brazil; b Institute of Environmental, Chemical and Pharmaceutical Sciences, Federal University of São Paulo, Diadema, Brazil; c Escola Paulista de Medicina, Universidade Federal de São Paulo, São Paulo, Brazil; d Centre for Medical Mycology in Latin America (CMM LATAM) Unit, São Paulo, Brazil

**Keywords:** Agricultural practices, Climate change, Environmental pollution, Host vulnerability, One Health, Veterinary mycology, Wastewater treatments

## Abstract

Antifungal resistance in Candida species has become a critical yet underrecognized public health challenge. While most efforts focus on hospital transmission and clinical antifungal use, growing evidence implicates environmental, animal-associated, and community niches in the emergence and spread of resistant Candida. Agricultural practices, ecosystem pollution, and wastewater contamination create selective conditions that can favor resistance beyond clinical exposure, while climate pressures reshape fungal adaptation and spread. This Opinion alerts the scientific and medical communities to the urgent need to broaden antifungal stewardship through a One Health lens, integrating environmental, clinical, and veterinary surveillance, policy and regulatory action, and attention to social determinants into resistance prevention. Addressing upstream ecological drivers is essential to contain the expanding threat of antifungal-resistant Candida and safeguard the effectiveness of current and future treatments.

## Highlights

• *Candida* antifungal resistance extends beyond hospital/clinical settings, emerging from environmental and community sources.
• Agricultural fungicides, ecosystem pollution, and climate pressures are implicated in shaping resistant *Candida* populations.
• Social and demographic factors amplify the burden, transmission and persistence of antifungal-resistant infections.
• Current hospital-centered mitigation overlooks upstream ecological, veterinary, and societal drivers of resistance.
• One Health must integrate environmental surveillance, regulation, and communityinterventions to curb antifungal resistance.

## 1 The expanding burden of antifungal resistance in Candida

Antifungal resistance in *Candida* species is a growing clinical and public health crisis often overshadowed by bacterial drug resistance [1], [2]. Invasive candidiasis and other *Candida* infections cause life-threatening disease in millions of people each year [3]. Globally, resistance patterns vary substantially across species. *Candida auris* (syn. *Candidozyma auris*) shows the highest rates, with fluconazole resistance frequently exceeding 80–90% and occasional multidrug resistance [4], [5]. For *Candida parapsilosis* global fluconazole resistance is around 17%, with regional variability and increasing trends [4]. Recent outbreaks of candidemia caused by fluconazole-resistant *C. parapsilosis* have been increasingly reported in Türkiye, India, China, and countries in Latin America and the Mediterranean region, with resistant strains accounting for 10–60% of all isolates [6]. For *Candida glabrata* (syn. *Nakaseomyces glabratus*)*,* fluconazole resistance rates generally range from 7 to 10%, although it reaches 15% in some regions. For *Candida tropicalis,* rates are typically 5–10% globally, but exceed 20% in some Asian countries. In contrast, *Candida albicans* remains largely susceptible to most antifungal classes, with global fluconazole resistance usually below 5%, although higher rates have been reported in certain middle-income regions [4].

Traditionally, the emergence of resistant *Candida* has been viewed primarily through two main factors, the overuse of antifungal drugs in human medicine and the acquisition of resistant infections in hospital environments [7], [8], [9]. Indeed, inappropriate or excessive consumption of antifungals in clinical and community settings, combined with suboptimal infection control in healthcare facilities, has contributed to selection and persistence of resistant strains. However, this view explains only part of the phenomenon. Mounting evidence indicates that *Candida*'s antifungal resistance problem results from an intricate interaction of clinical, environmental, animal and societal drivers that operate well beyond hospital walls and human consumption. In the environment, *Candida* is facing numerous selective pressures that are accelerating resistance in parallel to clinical and community use. In animals, antifungal-resistant *Candida* species are increasingly reported in livestock, companion animals, and wildlife, pointing to the role of animal-associated niches in the emergence and spread of resistance [10], [11], [12]. Furthermore, broader social vulnerabilities also amplify the burden of resistant infections [1], [2], [13], [14]. In other words, it's not just about hospital acquisition and human consumption, it's about a broader ecological and global context driving the emergence of resistant yeasts (Fig. 1).

**Fig. 1:**
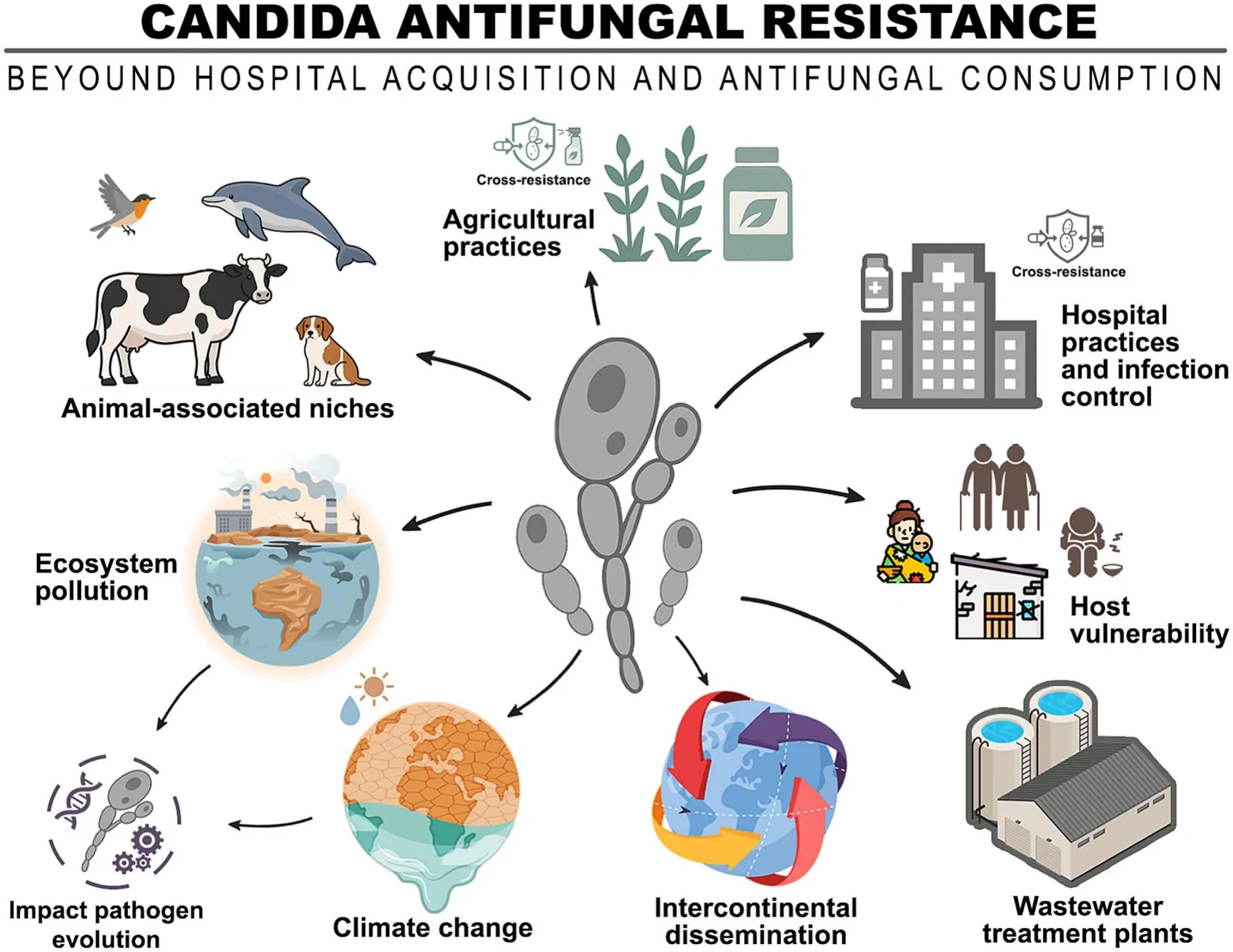
Drivers of antifungal resistance in *Candida* beyond clinical and community antifungal consumption. The diagram illustrates how resistance in *Candida* spp. is shaped by multiple interconnected drivers, including agricultural practices, animal-associated niches, hospital practices and infection control, ecosystem pollution, climate change, wastewater treatment plants, host vulnerability, and intercontinental dissemination. Notably, ecosystem pollution and climate change contribute to pathogen evolution, fostering adaptation and resistance development in *Candida* populations. Cross-resistance also emerges as a critical link, occurring in both agricultural and hospital contexts. The central illustration represents pathogenic *Candida* species, including both hyphae-forming and non-hyphae-forming yeasts, which differ in morphology but share resistance-driving mechanisms.

## 2 Environmental and climatic drivers of resistance

One under-recognized driver is the agricultural use of antifungals. Azole fungicides are heavily used in crop protection and agrochemical applications worldwide, and these chemicals are often structurally similar to medical azoles like fluconazole and voriconazole [15]. Recent studies have raised concern that such agricultural azole use exerts a selective pressure on environmental yeasts, potentially inducing cross-resistance to medical antifungals [16]. This phenomenon has already been well-documented in molds (*Aspergillus fumigatus* developing triazole resistance in fields sprayed with fungicides) [17], and now similar patterns are emerging for *Candida*. *Candida* species are not confined to humans; many pathogenic yeasts are ubiquitous in soil, water, and on plants [15]. Thus, antifungals applied on farms can directly affect these environmental *Candida* populations, positioning agricultural settings as key reservoirs and hotspots of antifungal resistance. A striking example comes from a study in Taiwan, where investigators found fluconazole-resistant *C. tropicalis* isolates in fruit orchards that were genetically nearly identical to drug-resistant strains causing human bloodstream infections [18]. Notably, patients infected with these strains had no recent azole exposure, implicating the farms, not the pharmacy, as the source of resistance. This epidemiological evidence suggests that intensive azole spraying in agriculture can seed reservoirs of resistant *Candida* in the environment, which then spill over into clinical and community settings. Laboratory science bolsters this link. Experimental exposure of *C. tropicalis* to an agricultural azole (tebuconazole) was shown to induce genomic changes (haploid formation) and cross-resistance to medical azoles in the yeast [19]. The authors emphasized that environmental application and accumulation of azoles could promote the evolution of antifungal resistance in nature. Selection pressures arising from agricultural practices may have downstream effects on clinical settings, contributing to the increasing prevalence of azole resistance in human infections.

Beyond fungicides, environmental and ecological factors at large are creating new opportunities for *Candida* to develop and spread resistance. *Candida* spp. can persist outside hospital settings, and emerging evidence suggests that changing ecosystems are selecting for more thermotolerant, drug-resistant strains [20]. *C. auris* is a paradigm of this dynamic. This multidrug-resistant yeast emerged almost simultaneously on three different continents in the past decade, an exceedingly unusual occurrence. Its sudden appearance in diverse regions with genetically distinct clades suggests a global environmental trigger rather than a single point-source outbreak. One provocative hypothesis is that climate change played a key role in *C. auris*' emergence [21], [22]. Rising temperatures favor thermotolerance and virulence traits, whereas extreme weather events, including droughts and floods, facilitate dispersal and the expansion of pathogenic fungi into new ecological niches, forcing them to adapt to rapidly changing environmental conditions [23]. *C. auris* may be the first example of a new fungal pathogen arising due to increased thermotolerance, enabling it to survive in human body temperature niches that were previously off-limits.

While climate change as a direct cause remains hypothetical, this opinion highlights how *Candida*'s evolution can be driven by environmental selective pressures beyond targeted antifungal use [13], [20]. Other ecological pressures, such as heavy metal pollution or exposure to industrial biocides in waterways, could similarly select for more robust, drug-tolerant yeast strains [20], [24]. Moreover, wastewater treatment plants, increasingly recognized as significant reservoirs of antifungal-resistant fungi, can further disseminate these organisms and resistance genes into natural ecosystems, thereby amplifying the environmental dimension of the *Candida* resistance crisis [25]. In essence, our changing environment is inadvertently conducting a grand experiment in fungal adaptation, and *Candida* is proving to be an adept test subject. Ultimately, climate change and ecosystem pollution are actively shaping pathogen evolution, driving *Candida* toward greater adaptability in diverse ecological contexts, disrupting the conventional hospital-centric view of antifungal resistance as a phenomenon restricted to healthcare environments.

## 3 Animal hosts and shared environments

Animal-associated niches are another under-recognized component of the *Candida* resistance problem. These yeasts colonize livestock, companion animals, and wildlife, and cause infections such as bovine mastitis, bringing their clinical relevance into animal production systems [26]. Resistance is already documented across these populations. In one study of 64*C. tropicalis* isolates from different animal species, 30 were resistant to fluconazole and 24 were resistant to both fluconazole and itraconazole [27]. Evidence from marine mammals further broadens this ecological picture, with resistant *C. albicans* recovered from cetacean respiratory samples [28]. Azole resistance has also been demonstrated in animal-derived isolates of *C. albicans*, *C. tropicalis*, and *C. parapsilosis* species complex [11]. Resistant *Candida* has been recovered from animals without previous antifungal treatment. This observation has prompted the hypothesis that exposure to fungicide residues through food, water, and soil can exert selective pressure on the animal microbiota, alongside the acquisition of resistant strains from contaminated environments [10]. Animal-associated resistance therefore brings the discussion back to the environmental pressures described above.

These animal-associated populations also connect the resistance problem to environments shared with people. *C. auris* has been recovered from dogs in India and from the oral cavity of a shelter dog in Kansas, USA. Genomic analysis of the Indian isolates showed relatedness to human clinical clade I strains, while the Kansas isolate was resistant to fluconazole and amphotericin B [29], [30]. In Italy, resistant *C. auris* was repeatedly recovered from the feces of a domestic cat over six months [31]. Wildlife extends this concerning ecological connection. In southern France, investigators identified shared *C. glabrata* genotypes in yellow-legged gulls and hospital patients, with fluconazole-resistant isolates present in both host groups. The feeding of these birds on human refuse and their movement between urban and coastal environments provide a framework for understanding how human activities, animal hosts, and environmental contamination can sustain interconnected yeast populations [12]. Animal populations can therefore contribute to a feedback loop in which resistant yeasts are acquired from shared environments and redistributed through shedding and contact. These connections place animal-associated niches within an ecological continuum linking environmental contamination, animal colonization, and the wider circulation of antifungal-resistant *Candida* [10].

## 4 Host and healthcare amplifiers

Host vulnerability, driven by aging, comorbidities and social vulnerability, amplifies the clinical impact of *Candida* and intersects with antifungal resistance dynamics [14], [32]. Immunosenescence, multimorbidity, antimicrobial exposure and frequent use of invasive medical devices in older adults increase colonization, candidemia risk, and mortality, thereby enlarging the pool of heavily treated hosts in whom resistant strains can be selected and sustained [32]. In parallel, poverty and related social determinants (overcrowding, poor housing, limited access to timely diagnostics and care, and higher environmental exposures) are consistently associated with greater fungal disease burden and delayed, suboptimal therapy, conditions that favor persistence and onward transmission of less susceptible *Candida* populations [33]. Long-term care and other resource-constrained healthcare settings, which disproportionately serve vulnerable groups, have repeatedly acted as reservoirs for *C. auris* and other drug-resistant yeasts, underscoring how host and system vulnerabilities compound environmental and pharmacologic selective pressures [34]. Recognizing host vulnerability is consistent with a One Health perspective. Beyond antifungal exposure, the burden and spread of resistant *Candida* depend on who is exposed, how promptly effective care is available, and where transmission persists.

Meanwhile, hospital practices and infection control measures contribute another piece to the resistance puzzle. *Candida* thrives in healthcare environments, especially when infection prevention is suboptimal [13]. In hospitals, these yeasts can persist on surfaces and medical devices, enduring routine disinfection. Reuse of inadequately sterilized equipment has caused outbreaks (for instance, a *C. auris* outbreak in a UK ICU traced to contaminated temperature probes) [35]. More insidiously, some *Candida* species are difficult to inactivate on contaminated surfaces using common disinfectants. *C. auris* again serves as a prime example and shows variable susceptibility to hospital biocides. Many strains are notably resistant to quaternary ammonium compounds like benzalkonium chloride, and can also survive less concentrated chlorine or chlorhexidine treatments [36]. This means that routine cleaning agents might leave behind *Candida* that can seed ongoing transmission. When disinfectants fail to fully eliminate the yeast, they exert a selective pressure similar to that of an antifungal drug, only the hardiest, often drug-resistant, cells remain. Compounding this issue is the frequent use of antiseptic agents (such as chlorhexidine baths for patients or triclosan in soaps) in healthcare settings [13]. Although these practices aim to reduce bacterial load, they may have unintended consequences for fungi. Experimental work has shown that prolonged exposure of *Candida* (*Nakaseomyces glabratus*, formerly *C. glabrata*) to sublethal doses of chlorhexidine or triclosan can induce cross-resistance to azole antifungals [37]. In the same study, dozens of *C. glabrata* isolates evolved decreased azole susceptibility after chronic antiseptic exposure, acquiring mutations in stress-response genes linked to drug resistance [37]. Thus, ubiquitous hospital biocides, from cleaning solutions to hand sanitizers, might be inadvertently training certain *Candida* populations to withstand antifungal drugs. This interplay of disinfectant tolerance and drug resistance blurs the line between infection control and antimicrobial stewardship: the products we rely on to sanitize hospitals could be co-selecting for drug-resistant strains.

## 5 Global dissemination and connected transmission networks

The global mobility of humans, animals, and goods ensures that *Candida* resistance issues are quickly shared worldwide [38]. A resistant strain that emerges in one corner of the world (whether in a patient, on a farm, or in nature) can hitchhike to new regions through travel and trade. Modern healthcare networks, with patients transferred across borders and medical tourism on the rise, have facilitated the spread of tough *Candida* strains [38], [39]. Within a decade of its emergence, *C. auris* was identified in over 40 countries spanning every inhabited continent. Similar patterns are seen with other resistant *Candida*; clonal strains of azole-resistant *C. tropicalis* and echinocandin-resistant *C. glabrata* have been reported in distant hospitals, suggesting intercontinental dissemination [40]. This global connectedness means environmental sources of resistance in one region (for example, azole-exposed agricultural fields or colonized wild birds) can eventually influence clinical infection outcomes in another region. Animal movement and trade belong in this global picture alongside human travel, because resistant yeasts also occupy the animals and environments connected by these networks [12], [30]. One Health is not just a slogan here but a reality: human, animal, and environmental health are intertwined in the story of *Candida* antifungal resistance.

## 6 Toward an integrated One Health response

In light of these multifaceted drivers, it becomes clear that fighting *Candida* resistance will require looking far beyond the prescribing practices of clinicians. As current evidence indicates, hospitals are not isolated environments but dynamic amplifiers within a larger ecological and epidemiological continuum. Individuals often enter healthcare facilities already colonized or infected by strains shaped by selective pressures operating in the community and the environment [13], [20]. Once inside, these strains encounter conditions that promote persistence, adaptation, and dissemination, creating a continuous feedback loop that links community and hospital settings. This bidirectional exchange challenges the conventional distinction between hospital- and community-acquired infections, revealing a shared resistance landscape maintained across both domains. Nevertheless, most mitigation strategies remain heavily hospital-focused [1], [41], prioritizing stewardship and infection control within healthcare walls while overlooking the upstream drivers that originate beyond them. Addressing antifungal resistance effectively will therefore require an integrated approach that bridges clinical, veterinary, community, and environmental fronts.

Antifungal stewardship in medicine remains crucial – we must curb unnecessary use and optimize dosing to minimize selection pressure in patients [41]– but it alone cannot solve the problem. What is the point of judiciously restricting fluconazole in the clinic if essentially the same azole compounds are being used en masse on crops and leaching into the soil and water? How can a hospital prevent resistant *Candida* infections if incoming patients (or even the hospital's water supply) already carry yeasts enriched in resistance genes from the outside world? These provocative questions underscore the need for a paradigm shift. We must expand our view to a One Health approach that tackles antifungal resistance on multiple fronts simultaneously. This means reexamining agricultural policies – for example, phasing out or regulating the farm use of azoles that select for medically relevant resistance, or at the very least preserving new antifungal treatments for human use before they are deployed in non-clinical settings. It also means improving environmental surveillance and veterinary diagnostics for pathogenic fungi, so we can detect and intercept resistant strains before they gain a foothold in vulnerable patient populations. Connecting these efforts through the integration of environmental monitoring, including wastewater surveillance, with clinical and veterinary surveillance would help identify shared resistance patterns and guide coordinated action. In animal production and care, this response should include veterinary antifungal stewardship, farm hygiene, and appropriate management of animal waste, complementing human stewardship within the same prevention strategy. Within healthcare settings, it means investing in infection control and cleaning protocols that account for hardy fungi: hospitals might need to use different disinfectants or higher concentrations to effectively kill organisms like *C. auris*. Alongside these preventive measures, advancing research and clinical implementation of antifungal combination therapies could help prolong the lifespan of current drugs and reduce the pace of resistance evolution. These efforts must also be supported by international cooperation to monitor and limit the spread of resistant strains via travel, much as is done for invasive bacteria.

*Candida* antifungal resistance is often called an emerging threat, but in truth it has been quietly building for years across the globe, fueled by our own activities in arenas far removed from the bedside. The rise of resistance is not happening in isolation within individual patients – it is happening in our farms, our hospitals, our communities, and our changing climate. A sobering lesson is emerging: every triazole sprayed on a tomato plant, every inadequately disinfected surface, and perhaps every degree of global warming can contribute to the evolution of a yeast that no longer responds to our drugs. Crucially, these pressures intersect with host vulnerabilities, aging populations, poverty, and other social determinants, that magnify the impact of resistance where resilience is weakest. The final burden of this resistance still lands on the critically ill patient battling an untreatable *Candida* infection, which is why we must act urgently. Combating this menace will require uncomfortable but necessary collaborations between clinicians, microbiologists, farmers, policymakers, and environmental scientists. It's time to broaden antifungal stewardship beyond the prescription pad. If we fail to recognize that it's not just about consumption – that *Candida* is resisting us not only because of what antifungal pills we swallow but also because of what we spray, flush, and transport – we risk losing the few effective antifungals we have left. The *Candida* resistance crisis is a symptom of a larger ecosystem problem, and only by addressing that larger problem can we hope to contain it. As we confront this challenge, we must be prepared to ask tough questions and make bold changes. After all, protecting human health in the era of antifungal resistance will demand nothing less than protecting the health of our farms, our environment, and our global community.

## CRediT authorship contribution statement

**Ronan Adler Tavella:** Writing – review & editing, Writing – original draft, Visualization, Software, Investigation, Formal analysis, Conceptualization. **Daniela Debone:** Writing – review & editing, Writing – original draft, Investigation, Formal analysis. **Simone Georges El Khouri Miraglia:** Writing – review & editing, Writing – original draft, Investigation, Formal analysis. **Arnaldo Lopes Colombo:** Writing – review & editing, Writing – original draft, Supervision, Investigation, Formal analysis, Conceptualization.

## Ethical approval

Not required.

## Funding

The authors thank the support from the 10.13039/501100001807Fundação de Amparo à Pesquisa do Estado de São Paulo (FAPESP) for the research grant awarded to the Antimicrobial Resistance Institute of São Paulo (ARIES, grant 2021/10599-3) coordinated by ALC, with SGEKM serving as an Associate Researcher, and for the postdoctoral fellowships to RAT (grant 2024/02579-0) and DB (grant 2024/02476-7); and the 10.13039/501100003593Conselho Nacional de Desenvolvimento Científico e Tecnológico (CNPq) for the Research Productivity scholarship to SGEKM and ALC (grant numbers 303245/2025-5 and 311372/2020-1).

## Declaration of competing interest

The authors declare that they have no known competing financial interests or personal relationships that could have appeared to influence the work reported in this paper.

## Data Availability

No data was used for the research described in the article.
